# Effects of Physical Rehabilitation With X-Sens Inertial Technology Feedback on Posterior Cerebral Artery Infarcts: A Case Study

**DOI:** 10.7759/cureus.56379

**Published:** 2024-03-18

**Authors:** Anisha K Sawra, H V Sharath, Nitika Chavan

**Affiliations:** 1 Department of Paediatric Physiotherapy, Ravi Nair Physiotherapy College, Datta Meghe Institute of Higher Education and Research (DU), Wardha, IND; 2 Department of Neurophysiotherapy, Ravi Nair Physiotherapy College, Datta Meghe Institute of Higher Education and Research (DU), Wardha, IND

**Keywords:** cimt, functional mobility, gait analysis, body weighted treadmill training, virtual reality, physiotherapy rehabilitation, stroke, posterior cerebral artery

## Abstract

Acute ischemic stroke (AIS) affecting the posterior cerebral artery (PCA) represents a unique clinical challenge, necessitating a multifaceted approach to rehabilitation. This review aims to provide a comprehensive overview of physiotherapeutic interventions tailored specifically for individuals with AIS involving the PCA territory. The PCA supplies critical areas of the brain responsible for visual processing, memory, and sensory integration. Consequently, patients with PCA infarcts often exhibit a distinct set of neurological deficits, including visual field disturbances, cognitive impairments, and sensory abnormalities. This case report highlights evidence-based physiotherapy strategies that encompass a spectrum of interventions, ranging from early mobilization and motor training to sensory reintegration and cognitive rehabilitation. Early mobilization, including bed mobility exercises and upright activities, is crucial to prevent complications associated with immobility. Motor training interventions target the restoration of functional movement patterns, addressing hemiparesis and balance impairments.

## Introduction

Cerebrovascular diseases, with stroke in their first place, are the most common neurological diseases of adults. They belong to chronic, mass non-infectious diseases. Stroke is an illness in which one or more blood vessels supplying the brain with oxygen and nutrients are damaged by a pathological process, and consequently, there is damage to the brain parenchyma [1]. Despite the obvious improvements in the prevention, diagnosis, treatment, and rehabilitation of persons with stroke, it still holds third place as the cause of death, after cardiovascular and malignant diseases. New studies based on an examination of the global burden of illness, the incidence, and death brought on by this disease worldwide also support these statistics [2].

Each interruption of blood flow (ischemia) to the brain means the discontinuation of oxygen and nutrient flow, and since nerve cells do not have a stock of nutrients, the disruption of blood flow leads to the cell's energy crisis. Ischemia can be global or regional, but an important point is the degree of ischemia compared to the normal flow and duration of ischemia. The higher the degree of ischemia and longer lasting, is more likely to occur irreversible changes which end in death (necrosis) of nerve cells [3]. There are two primary artery systems that provide blood to the brain: the anterior and posterior circulations.

The deep branches of the anterior and middle cerebral arteries (ACA and MCA) and the internal carotid artery (ICA) make up the anterior carotid circulation system. This confluence blood supplies nourishment to the orbit and most of the cerebral hemispheres, excluding the occipital lobe and a small area of the thalamus [4]. The vertebral artery, basilar artery, rear cerebral artery, and its branches make up the posterior circulation. They nourish the occipital lobe, a portion of the thalamus, the medio-inferior temporal lobe, and the majority of the brain stem [5].

The major objective of stroke patients' rehabilitation is to help them regain their social and personal identities as well as their maximal functional ability in everyday activities. For those over 60, stroke is the primary cause of rehabilitation as well as the primary source of functional disability [6]. Studies have shown that 10-20% of those who experience an ischemic stroke die somewhat soon after the stroke. The purpose of this study is to assess anterior circulation syndrome patients' functional recovery following their original ischemic stroke, the acute and post-acute phases of posterior circulation syndrome, and the chronic phase of physical therapy and rehabilitation [7].

## Case presentation

Patient information

The patient, a 44-year-old woman with a dominant right extremity, said she was unable to move her lower limb limbs or trunk and was taken to the hospital. She was too weak to walk, sit, or stand, had visual disturbances, and also had trouble doing activities of daily living (ADLs). A year prior, the patient suffered an ischemic stroke that left her with a quick onset of headache, difficulty speaking, and collapse from loss of consciousness. Seven days back, the patient started complaining of bilateral lower limb weakness, unable to sit, stand, or walk and decreased vision, slurred speech. The patient was immediately rushed to the hospital where investigations like CT brain and MRI brain were done which revealed chronic lacunar infracts involving bilateral corona radiata and ganglio-capsular region involved. The patient was admitted to the neuro ICU for 10 days and the patient was on 2 liters of O_2_ via nasal prongs, she was referred to neuro physiotherapy for further management, where the assessment was done, and according to the problem list, tailored physiotherapy rehabilitation was given.

Clinical finding

After admitting to the neuro ICU, the patient appeared unconscious, so a thorough examination was done. At first, mental state examination was not possible since the patient was unable to communicate. She was unable to speak or communicate. The inability to speak additionally impeded the sensory evaluation. Comprehensive evaluations were conducted on motor assessment, spasticity, and soft tissue compliance. Bilateral lower limb spasticity was graded 1+ (hypotonia), In the case of the shoulder, elbow, wrist, and hip flexors, and grade 3+ (hypertonia), in the case of the knee and ankle plantar flexors (Table 1).

**Table 1:** Reflexes (+): hypotonia; (++): normal; (++): hypertonia

Reflexes	Right side (affected)	Left side (non-affected)
Biceps	+	++
Triceps	+	++
Supinator	+	++
Knee	+++	+++
Ankle	+++	+++
Planter	+++	+++

The Tone grading scale was used to measure spasticity. The patient was unable to walk or stand. The relative gave a history of stroke two years back. She didn't use an assistance device to move regularly both inside her home and outside prior to having the stroke. The range of motion is important in tracking the progression or improvement of a condition, monitoring the effectiveness of interventions, or assessing the outcomes of rehabilitation, as shown in Table 2.

**Table 2:** Pre- and post-intervention range of motion

Joint	Pre-rehabilitation	Post-rehabilitation
Shoulder flexion	0-25°	0-150°
Shoulder extension	0-15°	0-30°
abduction	0-35°	0-120°
Medial rotation	0-35°	0-62°
Lateral rotation	0-35°	0-55°
Elbow flexion	0-45°	0-105°
pronation	0-45°	0-55°
supination	0-35°	0-50°
Wrist extension	0-35°	0-55°
Wrist flexion	0-35°	0-55°
Radial deviation	0-5°	0-15°
Ulnar deviation	0-10°	0-10°
Hip flexion	0-20°	0-160°
Hip extension	0-10°	0-35°
abduction	0-25°	0-135°
adduction	0-10°	0-25°
Knee flexion	0-30°	0-120°
Ankle planter flexion	0-25°	0-40°
Ankle dorsiflexion	0-5°	0-15°
Inversion	0-15°	0-35°
Eversion	0-5°	0-10°

Manual muscle testing (MMT) results helped in diagnosing muscle weakness or dysfunction. They provide information about the severity of muscle impairment, which is crucial for treatment planning, as shown in Table 3.

**Table 3:** Manual muscle testing (MMT) Grade 2: Full range of motion, gravity eliminated; Grade 3: Full range of motion against gravity; Grade 4: Full range of motion against gravity, moderate assistance

MMT	Pre-rehabilitation	Post-rehabilitation
Upper limb	Grade 2	Grade 4
Lower limb	Grade 3	Grade 4

Investigation findings

**Figure 1:**
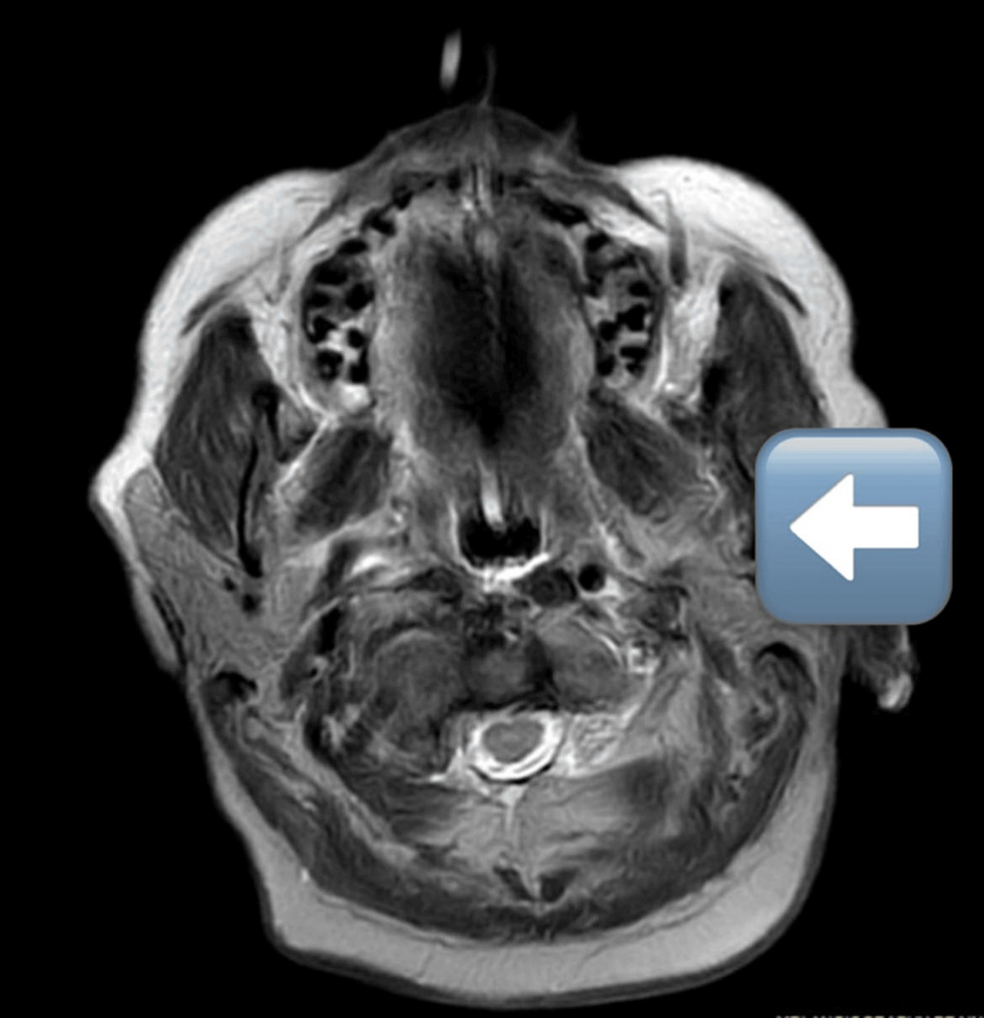
MRI brain impression reveals acute non-hemorrhagic infarcts involving bilateral cerebellum, dorsal pons, ponto-mesecephalic junction on the right side, and bilateral occipito-parietal cortex.

**Figure 2:**
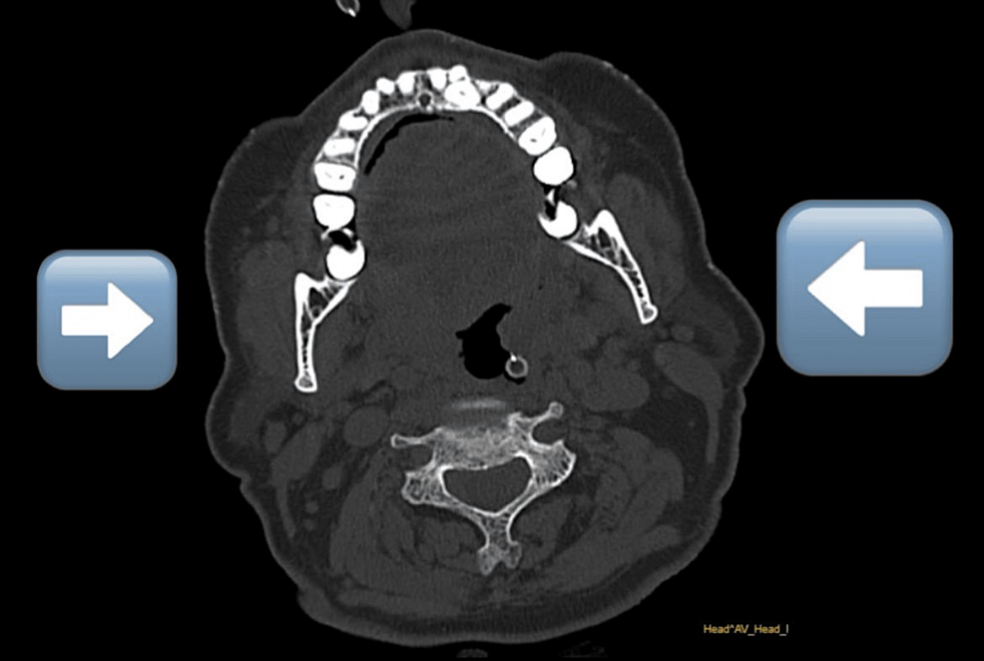
CT brain reveals diffuse irregular tortuous and narrow lumen with inhomogeneous signal seen involving the intracranial portion of bilateral internal carotid arteries.

The CT and MRI findings are consistent with posterior cerebral artery infarcts, providing crucial baseline data for assessing the effects of physical rehabilitation intervention with X-Sens Inertial Technology feedback. These imaging modalities offer insights into the extent of the infarct, associated complications, and potential areas for rehabilitation focus (Figure 1 and Figure 2).

Physiotherapy management

During the acute stage after stroke, the most predominant disturbances were arm muscle tone, strength, and balance; this phase lasted several days to weeks [8]. As the acute phase progresses to subacute, so does the development of spasticity. Active participation and mobility of the patient are strongly encouraged already in this phase. Gradual progression also begins first with sitting and finally with walking [9]. By the time, the patient reaches the chronic phase, postural and movement patterns, which were not completely treated during the previous phases, were treated with targets and problem list (Table 4 and Table 5) [10].

**Table 4:** Physiotherapy protocol for the acute stage CNS: Central nervous system

Sr	Intervention	Goals	Acute stage	Subacute stage	
1	Positioning	Provide Stable Base of Support , prevent further injury to affected limb, stabilizing body segments.	A pressure-relieving mattress, a foam or air-pressured mattress is used for positioning patients.	The patient’s limbs are placed into an antispastic position (extension of the upper limb, flexion of lower limb) and their distal parts are placed in a way that facilitates their functions. In all positions, the patient must be adequately stabilized with the main joints in the central position (Kolář, 2013).	
2	Respiratory muscle training	Lower dyspnea in order to enhance breathing patterns and increase the efficiency of respiration.	It is important to perform breathing exercises, facilitation of diaphragm, and promote proper clearance of the lungs to prevent any infections (Kolář, 2013).	Diaphragmatic breathing exercises were particularly beneficial, as they help improve the efficiency of the primary respiratory muscle.	
3	Reduce spasticity	To improve mobility, decreased pain, decreased muscle spasms, increased range of motion, improved positioning and prevention of contractures.	Gentle, repetitive movements of affected limbs by the therapist to maintain joint flexibility and prevent stiffness. Helps to reduce muscle tone and minimize contractures.	Targeted stretching of spastic muscles to improve flexibility. Rood faciliatory technique training for spasticity of muscles.	
4	Early mobilization	The goal of early mobilization and physiotherapy during this stage is to prevent complications, promote recovery, and improve overall functional outcomes.	Mild bed movement exercises aid in the prevention of joint stiffness, muscle contractures, and pressure sores. Exercises to enhance these areas are part of early rehabilitation to lower the chance of falls.	A crucial component of early mobility is encouraging ambulation, or getting the patient out of bed and moving about. In the beginning, the patient may need assistance with walking or the usage of walking aids.	
5	Swallowing dysfunction	To prevent risk of aspiration pneumonia	Coughing technique will be asked the patient to perform	Tongue movement will be initiated like, the pressing of the tongue against the palate to facilitate proper swallowing. Orofacial therapy improves motor re-education with sensory stimulation of the orofacial area. Treatment begins by positioning the patient with head in slight flexion to perform passive movements against spasticity.	
6	Balance training	To make patient functionally independent in daily living activities	Acute stage includes sit to stand with support, single leg balance, tree pose, side leg raises, standing with stationary march, seated march, pelvic bridging.	Sub-acute stage will be reaching for objects, weight shifting while standing, or practicing sitting-to-standing transitions, standing on uneven surfaces or incorporating head movements, can help improve balance. Lunges on stable and unstable surface and knee sitting to standing without support. Squats with wall support. Wall push-ups.	
7	Gait and mobility	To improve level of independence and strength	When necessary, support and enable safe walking by using assistive aids like canes or walkers. Take small, deliberate moves at first. equal distribution of weight on the lower limbs during stance. redistribution of the weight among the legs. Acceptance of heel strike and limb loading. Stance on one leg while maintaining control and stability. walking with obstacle training while using parallel bars.	Includes both lower and upper limb strengthening exercises, such as leg lifts, knee extensions, and resistance training with TheraBand’s or weights, ladder drills, cone drills, or stepping exercises, reaching for objects, trunk rotational reach-outs with single leg stance.	
8	Constraint Induced Movement Therapy	To increase an afflicted extremity's functional usage	Patient will perform exercises according to your ability with your affected hand for at least 10 to 20 minutes twice a day during the acute period of application.	Applied for two to three weeks, entailing the use of a padded mitt to immobilize the non-paretic arm for 90% of waking hours, task-oriented training with a high volume of repetitions for six hours each day, and behavioural techniques to enhance compliance and the patient's ability to transfer the activities from the clinical setting to their home.	
9	Body weighted Treadmill training	To improve strength, endurance and independent walking	For acute stage we performed treadmill for 5-6 minutes.	For sub-acute stage we progressed to inclined treadmill training for 10-12 minutes.	
10	Strength training	To improve balance function, mobility, and muscle strength of patients with stroke.	Isometric handgrip exercises, isometric knee extension while seated, assisted sit-to-stand exercises, assisted leg lifts, seated leg lifts, seated marching, seated bicep curls with light resistance, Seated leg press with a resistance band, seated rowing motion using a band.	thigh squeezes, or abdominal contractions, Seated leg lifts, seated arm raises, seated marching with 2-2.5 kg weight cuff, Seated leg press with a resistance band, seated row with a resistance band, Seated or supine pelvic tilts, hand grip strengthener exercises with resistance bands, scapular strengthening exercises, TheraBand’s exercises for shoulder flexors, abductors, on chair and for lower limb quadriceps, hamstrings. Dumbbell training for upper limb (1-2 kg)	

**Table 5:** Physiotherapy protocol for the chronic stage SP: Supine position; LP: Lying position; PP: Prone position

Sr	Goals	Intervention	Chronic stage rehabilitation
1	To improve gait ability, balance ability, stroke self-efficacy, and health-related quality of life in stroke patients	Robotic assisted training	The stroke patient is introduced to the robotic device, and the therapist ensures that the patient is comfortable and understands how to interact with the device. the robotic device guides the patient through a series of controlled and repetitive movements. The task included were reaching, grasping, lifting, or walking, depending on the goals of the therapy.
2	To help improve musculoskeletal system of patients with stroke	Vojta reflex locomotion	The program started with 5 daily sessions, for 1 week, each session is scheduled for 90 min, the program being carried out for a period of 6 months, from three positions: SP, LP, PP, activation time—5 min in each position, a total of 20 min of Vojta activation. Reflex Turning (Prone Position), Reflex Creeping (Supine Position), Rolling Reflex, Reflex Creeping on Hands and Knees, Reflex Lifting, Reflex Leverage (Side Position).
3	To help improve proprioceptive sensory changes in stroke patients	Sensorimotor training	Dysphagia- pharyngeal electrical stimulation, transcranial direct current stimulation and transcranial magnetic stimulation. For cognitive impairment and memory- includes the use of internalized strategies visual imagery, semantic organization, spaced practice and external memory assistive technology notebooks, paging systems, computers, other prompting devices). Exercise may be considered as adjunctive therapy to improve cognition and memory after stroke.
4	To improve motor function after stroke	Mirror therapy	Placing a mirror vertically in front of the patient. Wrist Extension and Flexion, Thumb Extension and Flexion, Inner Arm Stretch, Wrist Stretch, Stacking Coins, Pen Spin, Coin Drop, Finger Curl, finger scissor, finger pinch, finger grip, flat pinch, finger spread, finger extension, Shifting Exercise (Dexterity and Fine Motor Skills), Music Glove (Strength, Dexterity, and Fine Motor Skills).
5	VR enhances confidence, performance, motivation, and engagement to perform functional tasks through; Repetition of activities and sub-tasks, Increased sensory and visual feedback.	Virtual reality	VR was use for left side for patient perceives their affected side moving when it is really their unaffected side executing the movement. It helped in engaging environment for goal-oriented tasks for the patient. Patient had left side upper and lower limb weakness, using VR balance exercises, helping stroke survivors improve their stability and coordination.
6	To enhance speed, attention, and concentration	Augmented feedback	To check balance- we recorded the patient's movements and review the footage together, we got heel-off and circumduction gait. We taught the patient with tandem walking, keeping legs between the circles, obstacle training with augmented app, lower limb weakness impairment was easily achieved as regularly 3 hours a day was done.
7	To help regain their independence and improve their quality of life.	Vagus nerve stimulation	Singing, Humming, Chanting and Gargling exercises were given to patient.
8	To help improve forced-weight-bearing therapy to the lower limb	SPIDER programme	SPIDER device was used to actively stabilize the right knee joint as much as possible, in combination with the pelvic stabilization using elastic cords properties. Protocol also included wrist flexion and extension, finger flexion and extension, ankle dorsiflexion, and hip abduction/adduction as they were weak on right side of body. With time we increased resistance or repetitions to promote strength and endurance. Activities like fast walking, cycling, or rowing helped improved cardiovascular fitness and overall endurance. incorporated dynamic movements that involved multiple joints and muscle groups, this includes sit-to-stand exercises, walking with varied speeds, and reach-outs,
9	To increase strength, endurance	Aquatic therapy	Standing on the Bottom of the Pool, Walking and Associated Stepping Patterns, floating on the Surface, Floating prone, Floating supine, Breaststroke pull patterns performed at different depths with respect to the surface Underwater freestyle (alternating front crawl pull patterns without overarm recovery) degrees of abduction Formal swimming strokes can be introduced or reintroduced in the case of persons returning to training in the water, * Push/pull and/or sideways movements Forward/backward walking wall flutter-kick using a tray, Swimming and kicking in the prone and/or supine body positions, maintaining a neutral spine. Step-ups, mini squats, and single-leg balance Forward/backward walking - Marching wall flutter kick. Aqua-jogging Body position maintained with the aid of a floatation device Vertical bicycling, Vertical flutter kick, Vertical abduction/adduction,
10	To help improve joint mobility.	Contracture management	Gentle and prolonged stretching exercises can help to lengthen and relax tight muscles and tendons, Gentle joint mobilization techniques can be employed to improve joint flexibility and reduce stiffness. Applying heat to the affected area can help to relax muscles and increase tissue elasticity, making stretching exercises more effective. Warm packs.

Outcome measures

According to the functional assessment conducted by FIM, the patient mainly needed help with instrumental ADLs (communicating and handling medications) as well as fundamental ADLs (eating, washing, moving, and using the restroom) and all other outcome measures are mentioned in Table 6.

**Table 6:** Outcome measure taken pre and post treatment FIM: Functional Independence Measure; NIHSS: National Institutes of Health Stroke Scale; MoCA: Montreal Cognitive Assessment; MRS: Modified Rankin Scale

Sr	Scale	Pre treatment	Post treatment
1	FIM	45/126	105/126
2	MoCA	10/30	25/30
3	NIHSS	38/42	15/42
4	MRS	5/6	2/6

## Discussion

During the acute phase of stroke therapy early mobilization, positioning, ADL training, functional mobility training, and bed mobility are essential therapies. Mirror therapy is a method that has been demonstrated to help with mental health issues, post-stroke discomfort, and visuospatial neglect in addition to motor impairments [11]. During the sub-acute phase, the aim is to improve the patient's strength, balance, endurance, and mobility. Early overground bodyweight-support exercise is beneficial throughout the subacute phase. In the chronic stage, task-specific therapy was beneficial for a variety of motor deficits and impairments [12]. CIMT for upper limb impairment and motor function, robot-assisted training for upper limb function, obstacle walking, tandem walking, cardio-respiratory training, bilateral training for motor function of arms, mirror therapy for upper and lower limbs, Bobath, and early mobilization for mobility are some evidence-based rehabilitation treatments for motor recovery following a stroke [13,14]. There isn't just one activity that can aid in motor rehabilitation; rather, a variety of therapies can be used in conjunction with each other. This case study highlighted the importance of physical therapy treatments in stroke management and recovery while examining various rehabilitation strategies for the acute, subacute, and chronic phases of stroke recovery.

A posterior cerebral artery (PCA) infarct is a type of stroke that can lead to significant motor and balance impairments, impacting an individual's functional independence and quality of life. Physical rehabilitation plays a crucial role in promoting recovery and restoring motor function in patients with posterior cerebral artery infarct. Traditional rehabilitation approaches often rely on subjective feedback from therapists, which may limit the precision and effectiveness of interventions. In recent years, technological advancements, such as inertial sensors, have been integrated into rehabilitation programs to provide objective, real-time feedback to patients during therapy sessions. This case study investigates the effects of physical rehabilitation intervention incorporating X-Sens inertial technology feedback on motor function and balance in a patient with a PCA infarct.

This study aims to evaluate the effects of physical therapy, including input from XSENS inertial technology, on a patient who has a posterior cerebral artery infarct, as reported in a case report. The rare PCA type accounts for around 23% of all ischemic strokes; although symptoms vary based on the injured brainstem area, PCA-type stroke is associated with greater mortality than cortical hemisphere stroke. Lower extremities are more frequently affected by physical vital signs such as respiratory and circulatory failure [15]. One of the most common consequences of a stroke is loss of functional movement, which can significantly affect daily living because motor function is necessary for many daily tasks. Actually, more than 70% of stroke survivors report having trouble moving or using other brain processes [16-19]. 

Following the rehabilitation intervention incorporating X-Sens inertial technology feedback, the patient demonstrated significant improvements in motor function and balance. Scores on the FMA and MAS showed increased muscle strength, improved coordination, and enhanced motor control. Additionally, the patient exhibited better performance on the BBS, indicating improved static and dynamic balance. Posturography data revealed reduced sway and improved stability during standing and weight-shifting tasks [20].

Physical therapy has been demonstrated to enhance motor function, lessen disability, raise physical activity levels, and enhance quality of life. Additionally linked to structural brain remodeling, physical therapy helps stroke victims regain better motor function [21]. Thus, physical therapy plays a crucial role in the post-stroke care and rehabilitation process. The study's findings demonstrated the significance of task specificity and stroke severity in the rehabilitation of the upper and lower limbs following a chronic stage stroke. Functional outcomes increased dramatically after 16 weeks of therapy. Nonetheless, functional task practice and resistance strength training provided advantages right away [22].

An ideal XSENS sensor for rehabilitation is used which is sufficient to detect compensatory and dangerous movements, is widely accessible, affordable and has a low latency and a high sample rate (30-60 Hz). It also helped in counting the patient's characteristics, including the types and levels of impairments, and analyzing the patient's gait. Motion capture devices helped customize rehabilitation programs to target certain problems and enhance walking function, which gave extensive insights into gait anomalies in patients with stroke [23].

## Conclusions

The functional recovery of patients following acute ischemic stroke with the PCA is statistically significant during both the acute and post-acute phases of physical therapy and rehabilitation, according to the case report's findings. There was a noticeable improvement in both the overall and motor recovery following the stroke. A thorough, phase-by-phase therapy plan that assisted the patient in regaining basic ADLs was described. Regular exercise and focused teaching in gait, balance, and coordination helped patients' gait patterns; moreover, a rehabilitation program greatly raised the result marker.
